# The effect of the COVID pandemic lockdown measures on surgical emergencies: experience and lessons learned from a Greek tertiary hospital

**DOI:** 10.1186/s13017-021-00364-1

**Published:** 2021-05-07

**Authors:** Emmanouil Pikoulis, Nikolaos Koliakos, Dimitrios Papaconstantinou, Nikolaos Pararas, Andreas Pikoulis, Stavratis Fotios-Christos, Constantinos Nastos, Georgios Bagias, Eleni Boutati, Federico Coccolini, Fausto Catena

**Affiliations:** 1 3rd Department of Surgery, Attikon University Hospital, National and Kapodistrian University of Athens Medical School, Rimini Str. 1, 12462 Athens, Greece; 2 2nd Department of Internal Medicine-Propaedeutic, Attikon University Hospital, National and Kapodistrian University of Athens Medical School, Athens, Greece; 3 Emergency Surgery Unit & Trauma Center, Pisa University Hospital, Pisa, Italy; 4 Emergency and Trauma Surgery, Maggiore Hospital, Parma, Italy

**Keywords:** COVID-19, Emergency surgery, Emergency department, Hospital admissions

## Abstract

**Background:**

The COVID-19 pandemic caused a rise in healthcare demands leading to significant restructuring of hospital emergency departments worldwide. The aim of the present study is twofold: firstly, to discern any differences in regard to reason for surgical emergency department (SED) attendance and hospital admission during the pandemic and pre-pandemic eras in Greece, and secondly, to assess the impact of the lockdown measures implemented during the pandemic on SED patient attendance.

**Methods:**

Since the beginning of the COVID-19 pandemic in Greece (1 March 2020) and up to 15 December 2020, the charts of all adult patients arriving at the SED of the third surgical department of the “Attikon” University Hospital (a tertiary referral center for surgical and COVID-19 cases) were retrospectively reviewed and broken down in four periods reflecting two nationwide lockdown (period A 1/3/2020 to 30/4/2020 and period D 16/10/2020 to 15/12/2020) and two interim (period B 1/5/2020 to 15/6/2020 and period C 15/9/2020 to 30/10/2020) periods. Demographic and clinical data were compared to those obtained from the same time periods of the year 2019.

**Results:**

The total number of patients attending the SED decreased by 35.9% during the pandemic (from 2839 total patients in 2019 to 1819 in 2020). During the first lockdown, there was statistically significant reduction of motor vehicle accidents (*p*=0.04) and torso injuries (*p*=0.01). Contrarily, the rate of head injuries (*p*<0.001) and abdominal pain (*p*=0.04) were significantly increased. The same effect was observed regarding the rate of hospital admissions (*p*=0.002), although in terms of absolute numbers, admissions remained unchanged. During the second lockdown, there was a reduction in the number of perianal abscess cases (*p*=0.04) and hernia-related problems (*p*=0.001). An increase in the rate of fall injuries was also demonstrable (*p*=0.02). Overall, application of the lockdown led to a significant decrease in minor (*p*<0.001) and torso (*p*=0.001) injuries.

**Conclusion:**

The burden of the new COVID-19 disease has left a noticeable imprint in the function of emergency departments worldwide. In Greece, SED attendance was significantly reduced during the pandemic, an effect that was even more pronounced during the lockdown implementation; nevertheless, the overall rate of hospital admissions remained the same, denoting that patient care was not altered.

## Introduction

The new SARS-CoV-2 virus, commonly referred to as coronavirus disease 2019 (COVID-19), has been in the spotlight of the international medical community since its first appearance in December 2019. The worldwide spread of the disease and the severe mortality and morbidity associated with it has led the World Health Organization (WHO) to declare it as a pandemic in March 2020 [1].

Shortly after its emergence, the rapid spread of COVID-19 forced governments across the globe to implement unprecedented measures such as social distancing, avoidance of physical contact, and complete or partial confinement measures (colloquially termed as lockdown) in an effort to minimize contagion. The pandemic caused a rise in healthcare demands worldwide and has necessitated significant restructuring of hospital emergency departments. Importantly, COVID-19 has posed a significant strain on hospital systems largely due to the increase of hospitalizations of patients with coronavirus-related disease [2]. Most authorities opted to postpone elective surgical cases when feasible [3, 4] in an effort to relocate resources to COVID-19 patients. Nevertheless, the level of care for patients presenting with surgical emergencies necessitating urgent or emergent surgical interventions needed to be maintained [5]. Taking all these factors into account, it is plausible that the pandemic has drastically changed the patient synthesis of the surgical emergency department (SED) by limiting the number of patients seeking emergent surgical advice for potentially postponable causes. Moreover, we hypothesize that the severity of confinement measures (whether complete or partial lockdown) influences the type of emergencies that present in the SED.

The aim of the present study is twofold: firstly, to discern any differences in regard to reason for SED attendance and hospital admission between the pandemic and pre-pandemic eras, and secondly, to assess the impact of the lockdown measures implemented during the pandemic on SED patient attendance.

## Materials and methods

The charts of all adult patients (>18 years of age) presenting at the surgical emergency department (SED) of the third Surgical Department of the “Attikon” University Hospital (a tertiary university hospital and referral center) since the beginning of the COVID-19 pandemic in Greece (March 1, 2020) and up to December 15, 2020, were retrospectively reviewed in regard to their reason for presentation, the use of ambulance services, and the need for hospital admission. Admitted patients were further reviewed regarding the mortality rate, the length of hospital stay, ICU requirements, and the incidence of intra-abdominal infection. Patients using SED services during the same time period of the previous year (2019) were utilized as pre-pandemic controls.

During the pandemic, the Greek government imposed complete lockdown measures from 1/3/2020 to 30/4/2020, which prohibited civilians from leaving their home (with few exceptions, such as in the case of emergency), and partial lockdown measures from 16/10/2020 to 15/12/2020, which entailed a curfew starting from 9:00 pm to 6:00 am. No service-oriented businesses (such as bars or restaurants) were operating during any of the lockdown periods. Partial restrictive measures were applied from 15/9/2020 to 30/10/2020 which consisted of curfew starting from 12:00 am to 6:00 am with most service-oriented businesses operating normally in the meantime.

The entire patient cohort was broken down into four groups according to the timing of presentation; group A included patients from 1/3/2020 to 30/4/2020, group B from 1/5/2020 to 15/6/2020, group C from 15/9/2020 to 30/10/2020, and group D from 16/10/2020 to 15/12/2020. Group A coincides with the duration of the first lockdown in Greece, while group D coincides with the second lockdown effected during the second wave of the COVID-19 pandemic. Group B patients presented to the SED during a time period when no lockdown or restriction measures, whatsoever, were in effect and group C patients during a time period when partial restrictive measures (as described above) were applied. Patients in each of these groups were matched with patients visiting the SED during the exact same period during the year 2019. For each patient, a single chief complaint or reason for visiting the SED was registered.

All adult patients arriving at the SED in the designated time periods were eligible for inclusion. Cases involving consultation to other subdivisions of the emergency department and in-hospital referrals were excluded. Statistical analyses were performed using IBM SPSS Statistics, version 25 (SPSS Inc., Chicago, IL, USA). Non-parametric tests (Fischer’s exact test, Mann-Whitney *U*) were used to test for group differences. A *p*-value lower than 0.05 was considered statistically significant.

## Results

**Fig. 1:**
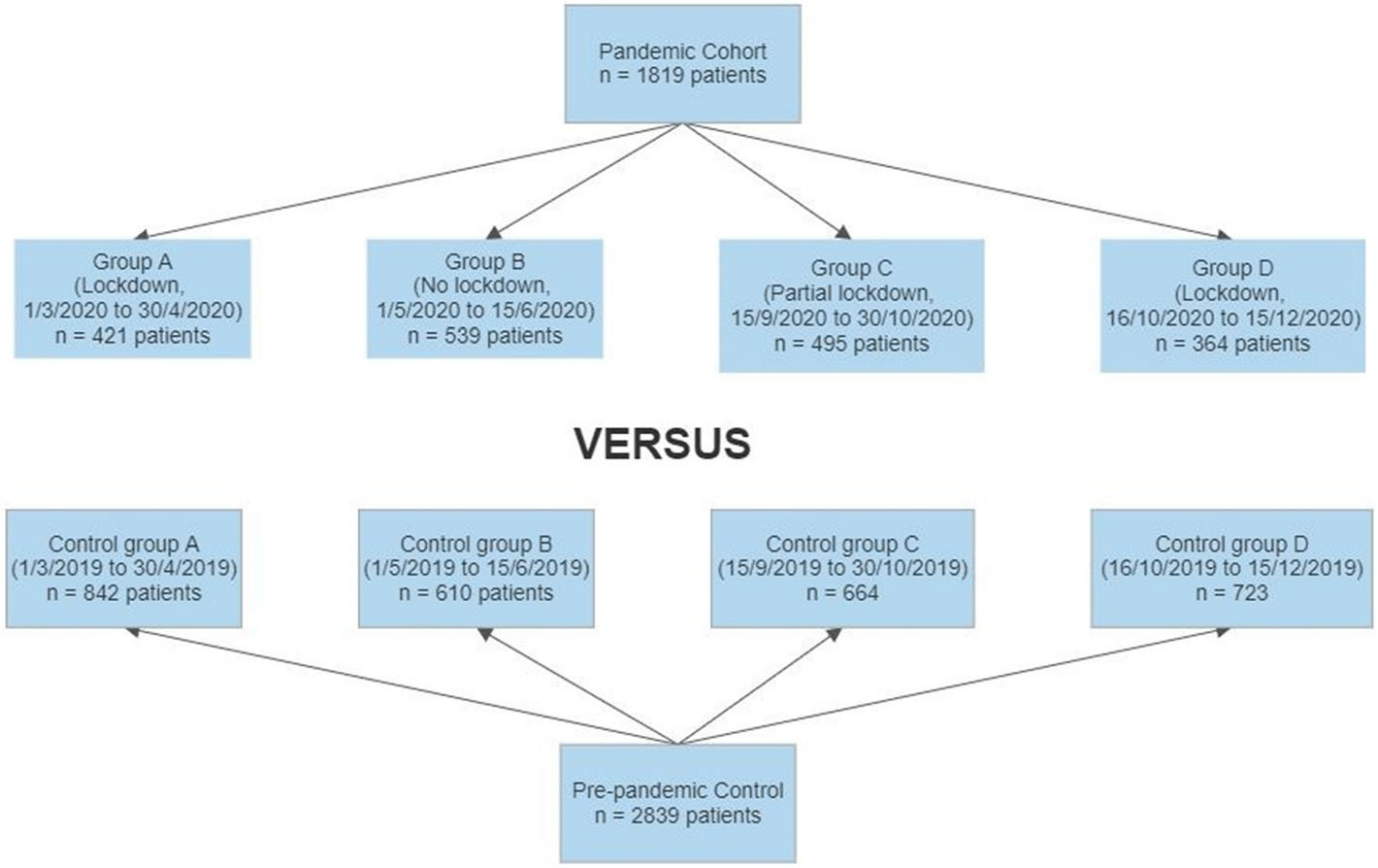
Flowchart of patient selection and breakdown of study periods

**Fig. 2:**
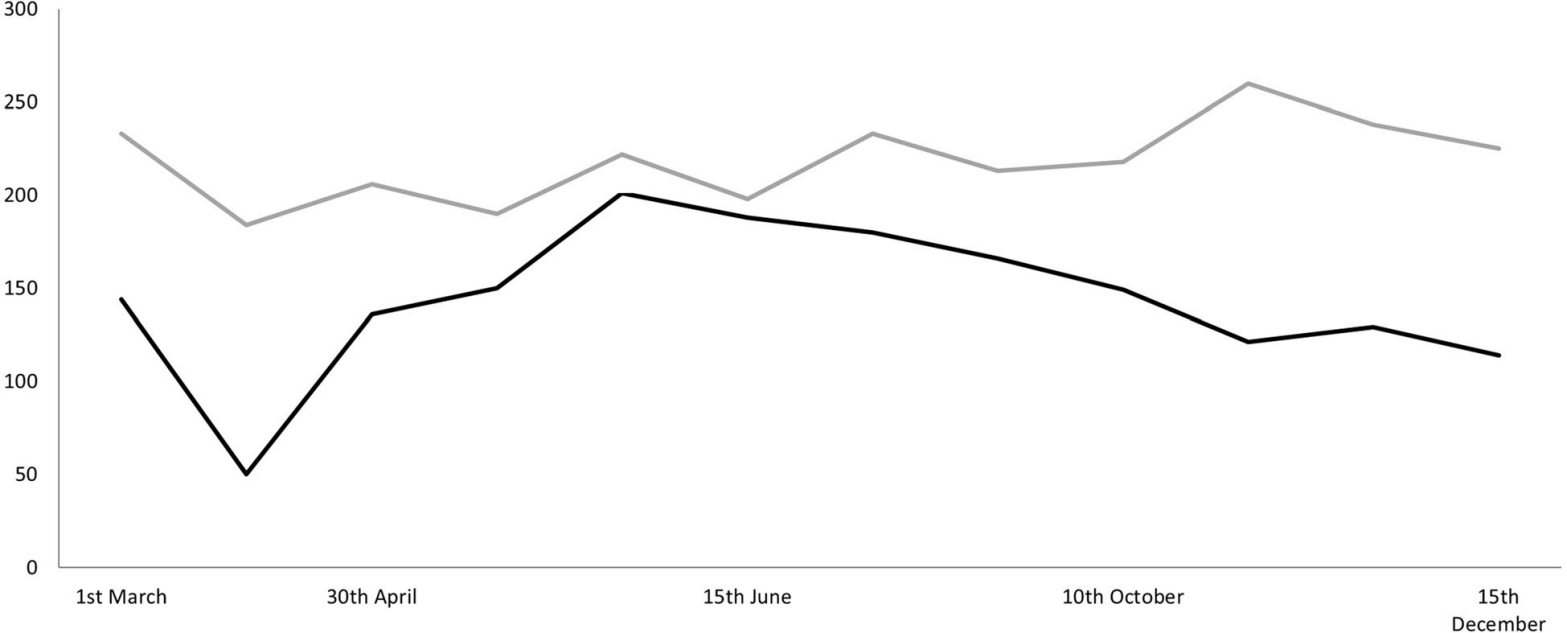
Surgical emergency department patients during and before the outbreak of the COVID-19 pandemic. Data are presented as absolute number of patients on the y-axis

In total, 1819 patients visited the SED during the designated study periods after the start of the pandemic. These patients were matched with 2839 patients that received care from the SED during the corresponding time periods of the year 2019. Figure 1 exhibits the flowchart of patient selection and Fig. 2 the trend of SED attendance, in total numbers, before and after the start of the pandemic.

**Fig. 3:**
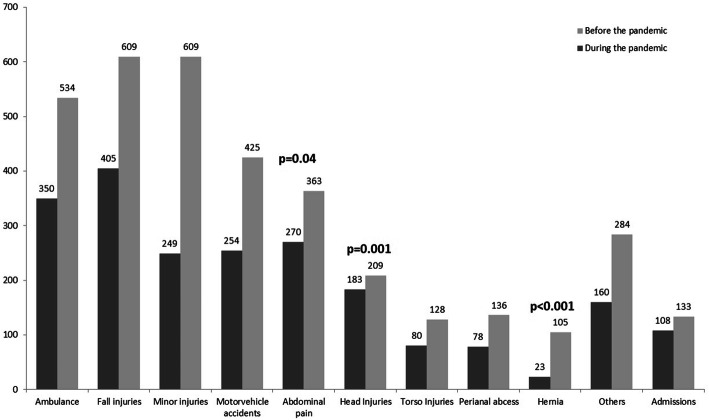
Reasons for visiting the surgical emergency department before and after the break out of the COVID-19 pandemic. Data are presented as number of patients on the y-axis

Regarding the reason for visiting the SED, fall injuries comprised the most commonly encountered cases both before (22.3%) and after the start of the pandemic (21.5%). During the study time period, there was a statistically significant reduction in patients seeking surgical attention for symptoms related to hernias compared to the pre-pandemic control group (1.3% vs. 3.7%, *p*<0.001). The rates of head injuries and abdominal pain were significantly elevated in the pandemic patient group (10.1% vs 7.4% and 14.8% vs 12.8%, respectively). Use of ambulance services before the arrival at the SED was equivalent between the compared groups. The admission rate was higher during the pandemic period (5.9% vs. 4.7% in the control group), albeit with no statistically significant difference. Figure 3 summarizes comparative patient data regarding the reason for visiting the SED.

During the initial lockdown (period A), a statistically significant reduction of motor vehicle accidents (10.5% vs 14.8%, *p*=0.04) and torso injuries (2.4% vs 5.2%, *p*=0.01), compared to the matched patient cohort during the same time periods of 2019, was observed. Concurrently, a significant rise in the rate of head injuries (11.4% vs 6%, *p*<0.001), abdominal pain (14% vs 12.4%, *p*=0.04), and hospital admissions (7.6% vs 3.7%, *p*=0.002) was registered. During time period D (second lockdown), a significant reduction in the cases of perianal abscess (2.2% vs. 4.7%, *p*=0.04) or hernia-related complaints (1.1% vs. 5.1%, *p*=0.001) was noticeable. A significant increase in the rate of fall injuries was also demonstrated (28.6% vs. 22.4%, *p*=0.02).

Analysis of patients presenting during time period B (lift of lockdown measures) did not reveal any statistically significant differences in reason for visiting the SED when compared to their 2019 counterparts. The same holds true for time period C (partial restrictive measures), except for hernia cases, which exhibited a significant reduction (1.6% vs. 5%, *p*=0.002). When patients presenting during the first lockdown (period A) were compared with those presenting during the second lockdown (period D), a significant reduction of perianal abscess cases was recorded in the latter group (5.9% vs. 2.2%, *p*=0.009). Patient data and comparisons are summarized in Table 1.

**Table 1:** Characteristics of patients visiting the surgical emergency department referral in the four different study time periods (A, first lockdown 1 March 2020 to 30 April 2020; B, 1 May 2020 to 15 June 2020, C, partial restrictions 15 September 2020 to 30 October 2020; D, second lockdown 16 October to 15 December 2020)

Time Period	A	***p***-value	B	***p***-value	C	***p***-value	D	***p***-value
Pandemic group vs control group (*n*, %)								
**Total ED patients**	421 vs 842		539 vs 610		495 vs 664		364 vs 723	
**Men**	254 (60.3) vs 490 (58.2)	0.48	294 (54.5) vs 365 (59.8)	0.07	302 (61) vs 367 (55.2)	0.05	220 (60.4) vs 387 (53.5)	**0.03**
**Women**	167 (39.7) vs 352 (41.8)	0.46	245 (45.5) vs 245 (40.2)	0.07	193 (39) vs 297 (44.8)	0.05	144 (39.6) vs 336 (46.5)	**0.03**
**Ambulance use**	83 (19.7) vs 168 (19.9)	0.92	100 (18.5) vs 116 (19)	0.84	101 (20.4) vs 116 (17.5)	0.2	66 (18.1) vs 134 (18.5)	0.87
**Fall injuries**	97 (23) vs 191 (22.7)	0.86	87 (16.1) vs 115 (18.9)	0.23	117 (23.6) vs 141 (21.2)	0.33	104 (28.6) vs 162 (22.4)	**0.02**
**Minor injuries**	57 (13.5) vs 129 (15.3)	0.51	78 (14.4) vs 94 (15.4)	0.65	63 (12.7) vs 90 (13.5)	0.68	51 (14) vs 102 (14.1)	0.96
**Motor vehicle accidents**	45 (10.7) vs 125 (14.8)	**0.04**	87 (16.1) vs 101 (16.5)	0.84	71 (14.3) vs 99 (14.9)	0.78	51 (14) vs 100 (13.8)	0.93
**Abdominal pain**	69 (14) vs 104 (12.4)	**0.04**	76 (14.1) vs 83 (13.6)	0.81	66 (13.3) vs 84 (12.7)	0.72	59 (16.2) vs 92 (12.7)	0.11
**Head injuries**	48 (11.4) vs 51 (6)	**<0.001**	50 (9.3) vs 44 (7.2)	0.2	54 (10.9) vs 57 (8.6)	0.18	31 (8.5) vs 57 (7.9)	0.71
**Torso injuries**	10 (2.4) vs 44 (5.2)	**0.01**	36 (6.7) vs 27 (4.4)	0.09	23 (4.6) vs 24 (3.6)	0.37	11 (3) vs 33 (4.6)	0.22
**Perianal abscess**	25 (5.9) vs 48 (5.7)	0.86	19 (3.5) vs 29 (4.8)	0.29	26 (5.2) vs 25 (3.8)	0.22	8 (2.2) vs 34 (4.7)	**0.04**
**Hernia**	4 (1) vs 19 (2.3)	0.1	7 (1.3) vs 16 (2.6)	0.1	8 (1.6) vs 33 (5)	**0.002**	4 (1.1) vs 37 (5.1)	**0.002**
**Others**	36 (8.5) vs 75 (8.9)	0.83	51 (9.5) vs 57 (9.3)	0.94	42 (8.5) vs 79 (11.9)	0.06	31 (8.5) vs 73 (10.1)	0.4
**Admissions**	32 (7.6) vs 31 (3.7)	**0.002**	33 (6.1) vs 32 (5.2)	0.64	21 (4.2) vs 42 (6.3)	0.12	22 (6) vs 28 (3.9)	0.1

**Fig. 4:**
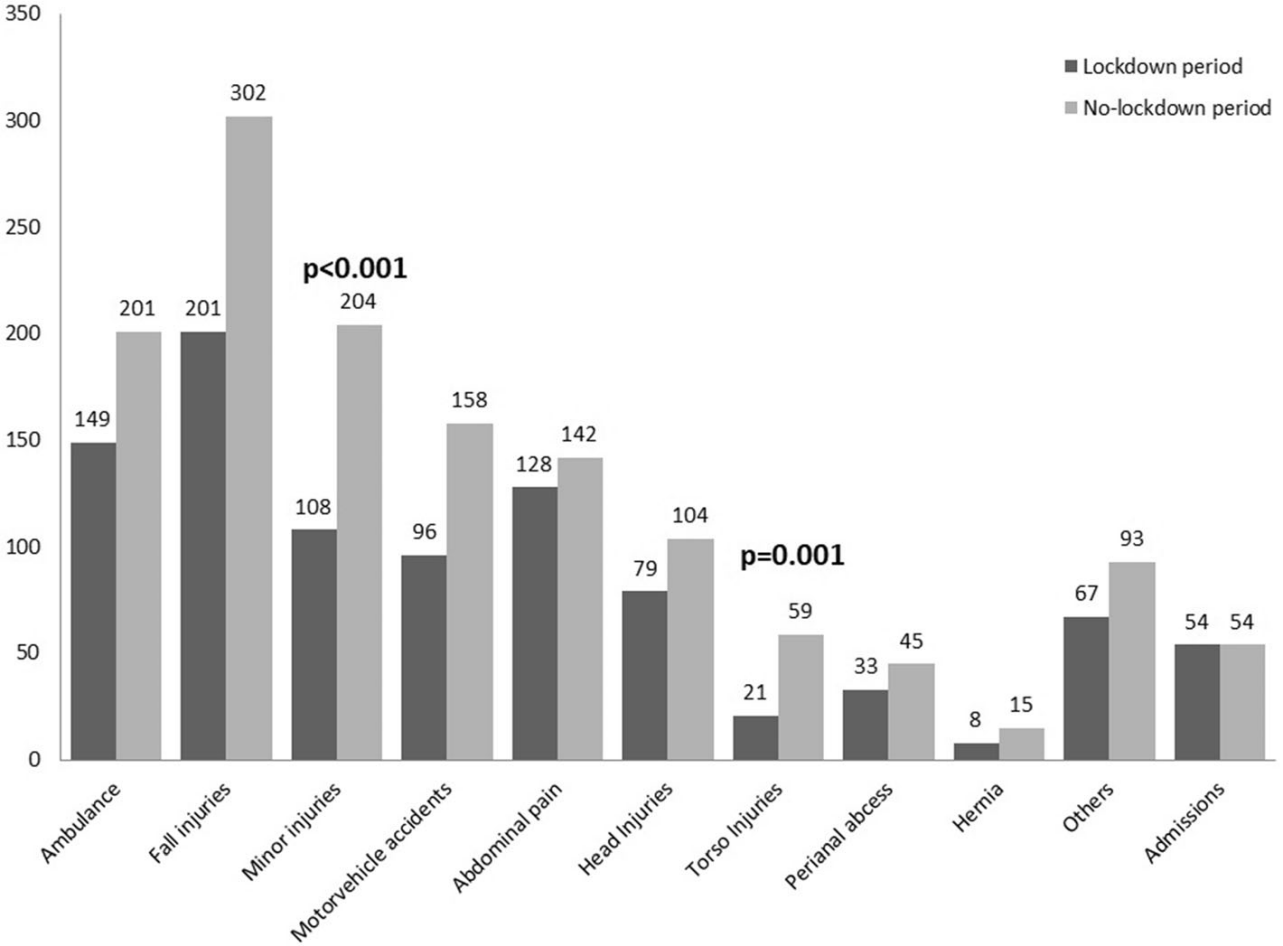
Reasons for visiting the surgical emergency department during the lockdown and no-lockdown periods of the pandemic. Data are presented as number of patients on the y-axis

**Fig. 5:**
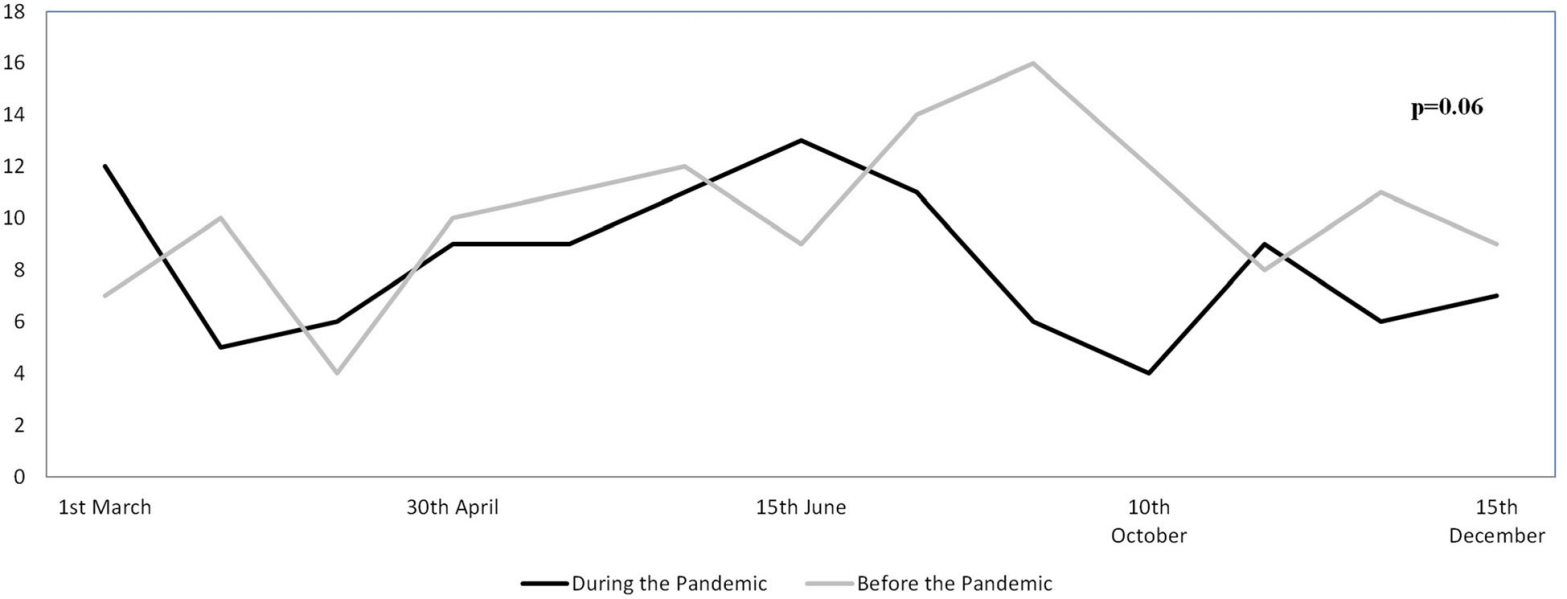
Total number of hospital admission from the emergency department

Further analysis of the lockdown and no-lockdown periods (A and D versus B and C) was undertaken (Fig. 4). There was a cumulative increase in SED attendance during the no-lockdown periods which translated in higher absolute numbers for each registered chief complaint. During the lockdown periods, there was a statistically significant reduction in minor injuries (13.7% vs 19.7%, *p*<0.0001) and torso injuries (2.7% vs 5.7%, *p*=0.001). The overall admission rate was slightly increased for the duration of the lockdown, albeit with no statistical significance (6.9% vs 5.2%, *p*=0.07). Of note, there was no statistically significant difference in ambulance use rates in any of the study time periods. Furthermore, the patient admission rate was significantly elevated only during time period A. When the entire patient cohort was compared with its pre-pandemic counterpart, no difference in the overall admission rate was observed (Fig. 5).

Charts of patients admitted to the hospital were further reviewed for each study time period separately (Table 2). There were no significant differences encountered in the admission rates for intra-abdominal infections, the ICU admission rate, mortality rate, or length of hospital stay in any of the study periods. In the entire patient cohort, only three cases of COVID-19-positive patients were encountered, all of which required admission on the basis of a surgical emergency.

**Table 2:** Admission-related parameters during the various phases of the pandemic. (A, first lockdown 1 March 2020 to 30 April 2020; B, 1 May 2020 to 15 June 2020; C, partial restrictions 15 September 2020 to 30 October 2020; D, second lockdown 16 October to 15 December 2020.) The exact same time periods of the year 2019 were used as controls

Time Period	A	***p***-value	B	***p-***value	C	***p-***value	D	***p-***value
Pandemic group vs control group (*n*, %)								
**Admissions for intra-abdominal infection**	6 (18.7) vs 5 (16.1)	0.27	7 (21.2) vs 5 (15.6)	0.58	4 (19) vs 9 (21.4)	0.82	5 (22.7) vs 7 (25)	0.84
**ICU admissions**	2(6.2) vs 3 (9.7)	0.61	4 (12.1) vs 3 (9.3)	0.35	2 (9.5) vs 3 (7.1)	0.74	0 vs 1 (3.6)	0.36
**Mortality**	3 (9.3) vs 2 (6.5)	0.42	3 (9) vs 2 (6.2)	0.66	3 (14.3) vs 2 (4.8)	0.18	2 (9) vs 1 (3.6)	0.41
**Length of hospital stay (mean ± SD)**	6.9±6.2 vs 11±17.9	0.39	6.8±6 vs 9.6±11.8	0.22	7±9.3 vs 7±6.7	0.91	6.3±4.2 vs 7.4±4.7	0.37

## Discussion

The COVID-19 epidemic is the first pandemic in the modern era, currently affecting most countries worldwide. Its rapid spread around the globe is unprecedented, involving many European countries like Italy [6], Spain [7], and France [8]. Following the example of the UK, many countries adopted a more lax strategy to fight the virus’ spread, but soon after implemented social distancing policies [9].

The Greek paradigm for combatting the spread of COVID-19 consisted of quick implementation of lockdown, shortly after the first cases of the disease were encountered within national borders (1st of March 2020), followed by relaxation of confinement measures after the first wave of the pandemic subsided (1st of May 2020). Parallel to social distancing measures applied to the general population, a nationwide foundational restructuring of emergency departments (a subset of which is the SED) occurred, as previously discussed. During this initial phase of the pandemic (which corresponds to time period A of our analysis), we observed a reduction in the overall attendance to the SED (421 total patients in 2020 versus 842 total patients in 2019) and significant reduction in the rates of motor vehicle accidents (10.7% vs 14.8%, *p*=0.04) and torso injuries (2.4% vs 5.2%, *p*=0.01). This finding stems directly from the curfew measures imposed, which effectively diminished motor vehicle traffic as well as outdoor assemblies which were a major cause for torso injuries (i.e., knife wounds, gunshot wounds, or cases of assault and battery). Simultaneously, we encountered an increase in the rates of head injuries (11.4% vs 6%, *p*<0.001), abdominal pain (14% vs 12.4%, *p*=0.04), and hospital admissions (7.6% vs 3.7%, *p*=0.002). This finding is likely illusory and may be attributed to the relative reduction in the remaining causes for SED attendance.

In fact, during the first half of the pandemic (time periods A and B), the absolute number of admissions was roughly equivalent to that of the previous year (Table 1, Fig. 5). Overall, 108 (5.9% of the cohort) admissions were made during the study period, compared to 133 (4.7%) in the control group. This finding denotes that appropriate hospital care was available throughout the pandemic, and more importantly, the fraction of patients with truly urgent or emergent surgical issues arriving at the SED was increased, despite the general reduction in SED attendance. This observation signifies that hospital resources were better allocated to those in need while simultaneously reducing the overall workload of the SED.

Interestingly, after resurgence of COVID-19 cases and the application of a second nationwide lockdown (corresponding to time period D of our analysis), a different pattern of causes leading to patient arrival at the SED was noted (Table 1). Motor vehicle traffic during this period was evidently higher than in the initial lockdown (plausibly due to intended or unintended laxity of governmental control), and consequently, no difference in motor vehicle accidents was observed when compared to the 2019 control group (14% vs 13.8%, *p*=0.93). Specifically, there was significant reduction in the cases of perianal abscesses (2.2% vs 4.7%, *p*=0.04) and hernia-related problems (1.1% vs 5.1%, *p*=0.001) implying that patients elected to postpone their visits to the SED or likely attempted to seek professional medical attention in an outpatient setting. Our analysis confirms that fall injuries are a major cause of SED attendance irrelevant of lockdown status.

Lift of lockdown measures in Greece occurred on the 1st of May 2020 (study period B). Immediately thereafter, the SED function returned to its pre-pandemic baseline, with a slight reduction in the overall attendance (11.6% reduction of serviced patients relative to the same period of 2019). In regard to patient demographics, reason for visiting, and admission rate, no statistically significant differences were noted when compared with the previous year (Table 1). The partial lockdown that followed later (starting from the 15th of September) led to a reduction in the total number of hernia cases addressed in the SED (1.6% vs 5%, *p*=0.002) without any other notable differences.

It thus becomes apparent that the type of patients that arrive at SED varies depending on the application of confinement measures. This phenomenon has been observed in other types of emergencies as well. Kuitunen et al. [10] in a large registry-based study in Finland observed a 16% decrease in emergency department visits during lockdown, with a considerable reduction in visits due to back or limb pain and infectious diseases. Similarly, Ojetti et al. [11] in a cross-sectional study of 16,281 Italian patients, derived an increased urgency of emergency department cases with a concomitant 15.2% increase in hospitalization rates. In our experience, the application of lockdown (whether partial or complete) led to a significant decrease of SED visits due to minor injuries (13.7% vs 19.7% in the no-lockdown group, *p*<0.001) and torso injuries (2.7% vs 5.7%, *p*=0.001), while the absolute number of patients attending the SED was reduced (Fig. 4).

Throughout the lockdown, evidently, patients avoided visiting the hospital on one hand due to fear of getting infected with the COVID-19 virus and on the other, so as to not overload an already stretched service. General practitioners must have played a significant role in treating patients with acute conditions, such as acute cholecystitis, with conservative treatment and phone follow-ups. De Simone et al. [12] reported that non-operative treatment could be applied in acute appendicitis, acute cholecystitis, adhesive bowel obstructions, and incarcerated hernias during the pandemic. East et al. [13] reported that manual reduction of incarcerated hernias under analgesia or sedation is a useful first line treatment in situations where surgical management is not immediately available.

In a subset analysis of patients with surgical emergencies, no significant differences were encountered in the admission rates for intra-abdominal infection, ICU admission rate, mortality rate, or length of hospital stay in any of the study periods (Table 2). This indicates that patients suffering from acutely presenting and unpostponable surgical emergencies continued to receive appropriate care irrelevant of applied confinement measures.

The main limitation of this study is its single-center retrospective design. The results may not be applicable to all hospitals. Many patients may have been admitted to less risky peripheral hospitals. Moreover, the after-effects of the reduction of SED patients cannot be accurately measured, and the overall impact it will bear on Greek national health system remains as yet unknown. Finally, annual fluctuations in surgical emergencies and trauma do occur, and therefore, the use of a patient cohort from 2019 as a pre-pandemic control group may not be an entirely accurate comparator.

## Conclusion

Ultimately, the COVID-19 pandemic has led to a review of national health systems around the world. Our experience demonstrates that the SED visits were significantly reduced during the pandemic. Nevertheless, this reduction can be credibly attributed to the implementation of lockdown measures and not solely on the pandemic itself. Mild emergencies such as hernias and perianal abscesses were mostly managed in an outpatient setting, while true emergencies received appropriate in-hospital care. Importantly, the total number of hospital admissions was decreased by only 18.8% during the pandemic, while the overall SED attendance was decreased by 35.9%. Simultaneously, the percentage of hospitalized patients was higher during the pandemic (5.9%) than the previous year (4.7%). This finding effectively demonstrates that patient care was maintained despite diversion of SED resources towards fighting the pandemic. The main issue that still remains unanswered to this date is whether neglected cases exist and are waiting to emerge after the end of the pandemic and what toll this may exact on the overall health status of the Greek population.

## Data Availability

The datasets used and/or analyzed during the current study are available from the corresponding author on reasonable request.

## Abbreviations

SED: Surgical emergency department
ICU: Intensive care unit
RT-PCR: Real time polymerase chain reaction
